# Supplementary material for: Searching for Osmosensing Determinants in Poplar Histidine-Aspartate Kinases
Source: Int J Mol Sci. 2023 Mar 28;24(7):6318. doi: 10.3390/ijms24076318 (PMC10093795; doi:10.3390/ijms24076318)
Supplement: Supplementary file 1 [file ijms-24-06318-s001.zip › Tab S1&S2.pdf]

Stresses	Concentrations	Osmolarity (milliosmol)
NaCl	0.3 M	841 ± 5.52
	0.6 M	1480 ± 4.26
	0.9 M	2110 ± 10.70
Infused PEG	50% (m/v)	1024 ± 17.72
Pure PEG	50% (m/v)	1393 ± 61.71

Table S1: Osmolarity measured for different stress media with NaCl and PEG.
Results are mean of 3 repeats.

Mutants	Primers	Sequences
HK1a-T324A	HK1a-T324A For<br>HK1a-T324A Rev	CGTAGGTGTTgCTACTGCTCT<br>ACGGCCACGATGCTTTTATTG
HK1a-S304A	HK1a-S304A For<br>HK1a-S304A Rev	CCACTGCTTgCAGCAGCATT<br>TGAATCCGTGTACTTGCTTACTGC
HK1a-K296A	HK1a-K296A For<br>HK1a-K296A Rev	GCAGTAAGCgcGTACACGGAT<br>CACATGCCATGAAGCCACA
HK1a-T324A-S304A	HK1a-S304A For<br>HK1a-S304A Rev	CCACTGCTTgCAGCAGCATT<br>TGAATCCGTGTACTTGCTTACTGC
HK1a3M	HK1a-K296A For<br>HK1a-K296A Rev	GCAGTAAGCgcGTACACGGAT<br>CACATGCCATGAAGCCACA
HK1b-T331A	HK1b-T331A For<br>HK1b-T331A Rev	TGTAGGTGTTgCTACTTCCCTCT<br>ACGGCCACGATGCTTTTATTG
HK1b-Q311A	HK1b-Q311A For<br>HK1b-Q311A Rev	CCTCTGCTTgcAGCAGCATTG<br>TGAATCTGTATACTTGCTTACTGCCAC
HK1b-K303A	HK1a-K303A For<br>HK1a-K303A Rev	GCAGTAAGCgcGTATACAGATTC<br>CACATGCCATGAAGCCACA
HK1b-T331A-Q311A	HK1b-Q311A For<br>HK1b-Q311A Rev	CCTCTGCTTgcAGCAGCATTG<br>TGAATCTGTATACTTGCTTACTGCCAC
HK1b3M	HK1b-K303A For<br>HK1b-K303A Rev	GCAGTAAGCgcGTATACAGATTC<br>CACATGCCATGAAGCCACA
HK1a-ECD1b	HK1ECD For<br>HK1ECD Rev	GCTTCTGGCCTTCGATATGAA<br>GGAATGATGATCACCCCCAC
HK1a-F470C	HK1a-F470C For<br>HK1a-F470C Rev	ggatgtgtctGcattttgattc<br>gatgactaggatacatagagatgc
HK1a2M	HK1a-F470C For<br>HK1a-F470C Rev	ggatgtgtctGcattttgattc<br>gatgactaggatacatagagatgc
HK1b-ECD1a	HK1ECD For<br>HK1ECD Rev	GCTTCTGGCCTTCGATATGAA<br>GGAATGATGATCACCCCCAC
HK1b-C478F	HK1b-C478F For<br>HK1b-C478F Rev	tggatgtgtctTcattttgattc<br>TTAGTGAGAATCAAAATGCAGAC
HK1b2M	HK1b-C478F For<br>HK1b-C478F Rev	tggatgtgtctTcattttgattc<br>TTAGTGAGAATCAAAATGCAGAC

Table S2: Primers used for point and domain-exchange mutants for HK1a and HK1b
