# Supplementary figures and images for: Searching for Osmosensing Determinants in Poplar Histidine-Aspartate Kinases
Source: Int J Mol Sci. 2023 Mar 28;24(7):6318. doi: 10.3390/ijms24076318 (PMC10093795; doi:10.3390/ijms24076318)

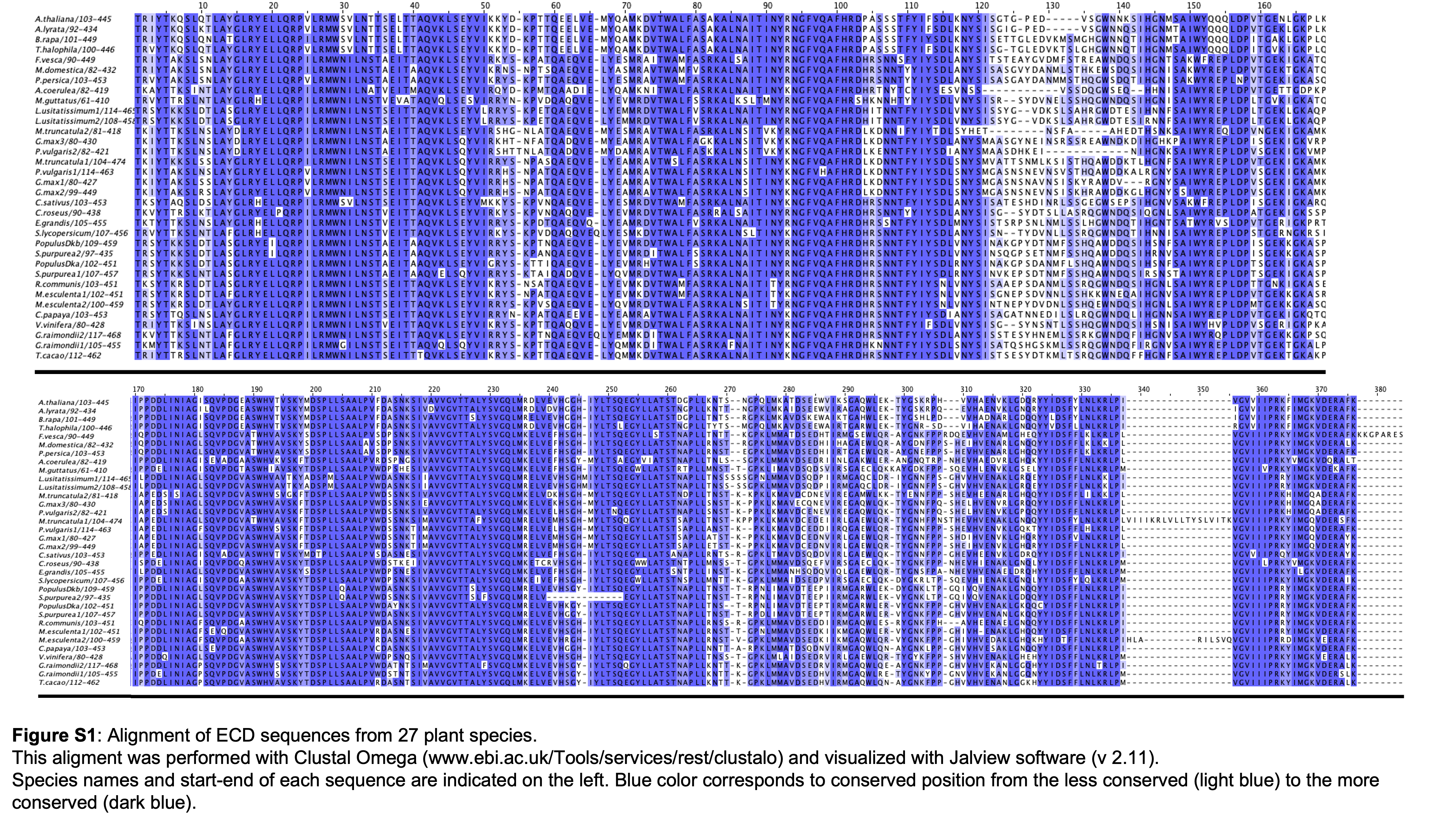

Supplement: Supplementary file 1 [file ijms-24-06318-s001.zip › Fig S1.png]
